# Heat of Vegetables and Animals

**Published:** 1852-01

**Authors:** 


### HEAT OF VEGETABLES AND ANIMALS.

After the experiments of John Hunter on the calorific function of animals and plants, it was generally esteemed a point settled in physiology, that life was incompatible with freezing—that to freeze was to kill them.

Late researches seem to render the truth of this conclusion doubtful, or rather to prove that this is untrue as to many plants, and a large section of the animal kingdom. The warm-blooded vertebrata are certainly destroyed by freezing, and the same is the fact with regard to many insects in their perfect state; but fishes and reptiles are often frozen with impunity. Prof. Kirtland, of Cleveland, has seen eels revive after having lain in a frozen condition for twenty-four hours. Prof. Lathrop, in the *American Journal of Science and Arts,* for this month, remarks, that "it is a fact well known to those who are accustomed to take fish, such as the common perch, the lake mullet, &c., from Lake Champlain in the winter, that these fish may be frozen perfectly solid and be transported many miles and kept several days, when upon thawing them out in a tub of cold water, they will be found to be alive and active. I find the same fact sustained in the case of the buffalo fish taken from the Rock river at this place." Sir John Franklin and other Arctic voyagers relate, that certain species of fish which are found imbedded in the ice of the polar regions, are restored to life when thawed. In a paper by Prof. Le Conte in the journal just referred to, it is stated, that "Hearne in his journey from Hudson's Bay to the Northern Ocean, found various species of frogs so completely frozen that their legs were as brittle as pipe-stems, and which resumed their natural movements when exposed to a genial heat." Hearne adds, that if the frogs be frozen a second time they invariably perish. The same traveler found grubs, and spiders in a frozen condition, with similar recuperative powers on exposure to a warm atmosphere. Carpenter, in his General and Comparative Physiology, quotes numerous instances of a like character; as of "caterpillars so frozen that when dropped into a glass they chinked like stones, but nevertheless revived" on being thawed. The following experiment was tried in Sir John Ross's voyage, and is conclusive on this point. Thirty larvæ of a caterpillar were put in a box and exposed to the winter temperature for three months; on bringing them into the cabin, every one of them returned to life and crawled about. They were again exposed to an atmosphere 40 deg. below zero, and instantly became re-frozen; after a week they were brought again into the cabin, and twenty-three returned to life. These were again exposed and re-frozen; and, after being solid for another week; eleven of them recovered on being brought into the cabin. A fourth time they were frozen, and only *two* of them recovered.

Prof. Le Conte has made observations on plants in a cold atmosphere and has arrived at results similar to the above. He has proved by numerous experiments that the cabbage, the rose, and many other plants may be hard frozen without serious injury. But such facts are familiar to every observer.

As to the temperature-preserving power of vegetables, it is now proved, contrary to the conclusions of the immortal Hunter, that vitality is but little concerned in the process. If the interior of large trees is found to be warmer in winter than the external atmosphere, it is because the tree is in some degree protected by its integuments from the cold, and derives its sap from the comparatively warm soil below.

